# Study of the potential neuroprotective effect of *Dunaliella salina* extract in SH-SY5Y cell model

**DOI:** 10.1007/s00216-021-03819-1

**Published:** 2021-12-18

**Authors:** Rocío Gallego, Alberto Valdés, José David Sánchez-Martínez, Zully J. Suárez-Montenegro, Elena Ibáñez, Alejandro Cifuentes, Miguel Herrero

**Affiliations:** Laboratory of Foodomics, Institute of Food Science Research (CIAL, CSIC-UAM), Calle Nicolás Cabrera 9, 28049 Madrid, Spain

**Keywords:** Alzheimer’s disease, *Dunaliella salina*, Neuroprotection, Carotenoids, Aβ1-42, Glutamate

## Abstract

Alzheimer’s disease (AD) is the most common form of dementia caused by a progressive loss of neurons from different regions of the brain. This multifactorial pathophysiology has been widely characterized by neuroinflammation, extensive oxidative damage, synaptic loss, and neuronal cell death. In this sense, the design of multi-target strategies to prevent or delay its progression is a challenging goal. In the present work, different in vitro assays including antioxidant, anti-inflammatory, and anti-cholinergic activities of a carotenoid-enriched extract from *Dunaliella salina* microalgae obtained by supercritical fluid extraction are studied. Moreover, its potential neuroprotective effect in the human neuron-like SH-SY5Y cell model against remarkable hallmarks of AD was also evaluated. In parallel, a comprehensive metabolomics study based on the use of charged-surface hybrid chromatography (CSH) and hydrophilic interaction liquid chromatography (HILIC) coupled to high-resolution tandem mass spectrometry (Q-TOF MS/MS) was applied to evaluate the effects of the extract on the metabolism of the treated cells. The use of advanced bioinformatics and statistical tools allowed the identification of more than 314 metabolites in SH-SY5Y cells, of which a great number of phosphatidylcholines, triacylglycerols, and fatty acids were significantly increased, while several phosphatidylglycerols were decreased, compared to controls. These lipidomic changes in cells along with the possible role exerted by carotenoids and other minor compounds on the cell membrane might explain the observed neuroprotective effect of the *D. salina* extract. However, future experiments using in vivo models to corroborate this hypothesis must be carried out.

**Graphical abstract:**

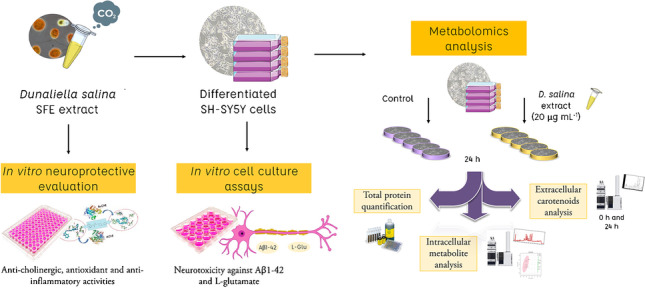

**Supplementary Information:**

The online version contains supplementary material available at 10.1007/s00216-021-03819-1.

## Introduction

Alzheimer’s disease (AD) is the most common form of dementia which is provoked by a progressive loss of neurons from different regions of the brain [1], causing loss of cholinergic neuronal activity, cognitive decline, and mental deterioration. There is not a specific origin for the severe neurodegeneration caused by AD. Complex and still not fully understood relationships among genetic, environmental, and nutritional factors; oxidative stress; and neuroinflammation might lead to the occurrence of this disease.

Two of the main neuropathological hallmarks in AD are the presence of intracellular neurofibrillary tangles (NFT), an abnormally phosphorylated form of protein tau, and the undesirable formation of amyloid-beta (Aβ) plaques outside neuronal cells, which are formed by an abnormal accumulation of insoluble Aβ peptides. The aggregation of these Aβ plaques leads to severe neurotoxic effects, disrupting the signaling process and triggering inflammation, functional losses, and other important damages [2]. Another molecule involved in AD progression is glutamate, which is an important excitatory neurotransmitter of the central nervous system. This molecule contributes to the normal neural transmission but its excessive accumulation leads to uncontrolled depolarization of neurons, causing excitotoxicity and neuronal death. These increased levels have been associated with several neurodegenerative diseases, including AD and Huntington’s and Parkinson’s diseases [3].

Up to now, there is no cure for AD and current therapies are only focused on the alleviation of symptoms but they are not able to stop or reverse the progression of the illness. However, some preventive methods such as the modification of lifestyle factors have been proposed for slowing its occurrence. Diet is one of the most relevant factors that can affect global health, and the role of a healthy nutrition in the reduction of AD risk has been extensively described [4, 5]. In this sense, there is a large amount of natural bioactive compounds that can interfere with different molecular mechanisms related to AD development. These dietary components include phenolic compounds, omega-3 fatty acids, fat-soluble vitamins, isothiocyanates, or carotenoids [6].

Among these natural constituents, carotenoids are lipophilic molecules mainly found in natural sources such as plants, seaweeds, and microalgae. These natural pigments have been associated with an extensive list of potential health benefits, mainly related to antioxidant or free radical protectors. However, new findings have confirmed that some of these bioactive compounds also have neuroprotective potential, even showing improvements in learning cognitive functions [6]. Furthermore, an interesting study published by Mullan et al. [7] showed that the serum levels of six carotenoids (lutein, zeaxanthin, β-cryptoxanthin, α-carotene, β-carotene, and lycopene) were significantly lower in patients with AD compared with cognitively intact controls [7]. This fact underlines the importance of acquiring a deeper understanding about the mechanisms of action related to the dietary intake of these compounds in AD development.

The inhibition of cholinesterase enzymes has also a key role in AD progression, since it helps to block the normal breakdown of acetylcholine, the main neurotransmitter in the brain. Specifically, acetylcholinesterase (AChE) inhibitors have been related to the reduction and prevention of the formation of Aβ plaques, protecting neurons from neurodegeneration. In this regard, a recently published study from our group has evaluated the in vitro inhibitory AChE activity of several carotenoid-enriched extracts from the microalgae *Dunaliella salina*, showing promising neuroprotective potential [8].

The study of the effect of any potential drug or extract needs to be validated through in vitro and in vivo models before accomplishing any human clinical trial. In this sense, cell cultures, specifically the SH-SY5Y cells, are cheap and easily cultured, and they can be used to evaluate the neuroprotective potential of different compounds. SH-SY5Y cells present a mature neuron-like phenotype when differentiated with retinoic acid (RA) and brain-derived neurotrophic factor (BDNF), simulating the early-stage pathophysiology of cholinergic neurons affected by AD [9]. Previous works have analyzed the lipidomic composition of this cell line [10] and, more recently, the potential changes in mitochondrial phospholipid classes of SH-SY5Y transfected cells to simulate an AD model [11]. Moreover, the effect of an olive extract on these cells has also been evaluated in terms of lipid alterations [12]. However, a comprehensive metabolomics study including the analysis of polar and non-polar compounds of these neuron-like cells treated with carotenoids-enriched extract has never been accomplished.

The main goal of the present work is to assess the potential neuroprotective effect of a carotenoid-enriched extract from *D. salina* microalgae obtained by supercritical fluid extraction, using different in vitro assays including antioxidant, anti-inflammatory, and anti-cholinergic activities, together with neuroprotection evaluation against two neurotoxic agents in the human neuron-like SH-SY5Y cell model. Moreover, a comprehensive metabolomics study based on the use of charged-surface hybrid chromatography (CSH) and hydrophilic interaction liquid chromatography (HILIC) coupled to high-resolution tandem mass spectrometry (Q-TOF MS/MS) was applied to evaluate the effects of the extract on the metabolism of these cells.

## Materials and methods

### Samples, chemicals, and reagents

Freeze-dried *D. salina* microalgae biomass was kindly supplied by A4F—Algae for Future (Lisbon, Portugal) and stored in the dark at − 20 °C in the absence of oxygen until use. For the supercritical fluid extraction, premier quality CO_2_ was provided by Carburos Metálicos (Madrid, Spain).

Chemical reagents AChE from *Electrophorus electricus* (electric eel) type VI-S, butyrylcholinesterase (BChE) from equine serum, and 2,2-azino-bis (3-ethylbenzothiazoline-6-sulphonic acid) (ABTS) were purchased from Sigma-Aldrich (Madrid, Spain), together with Trizma hydrochloride, bovine serum albumin (BSA), (KH_2_PO_4_) ≥ 99.0%, (NaH_2_PO_4_) ≥ 99.0%, fluorescein sodium salt, quercetin, linoleic acid (LA), ( ±)-6-Hydroxy-2,5,7,8-tetramethylchromane-2-carboxylic acid (Trolox) > 97%, acetylthiocholine iodide ≥ 99.0%, and lipoxygenase (LOX) from glycine max (soybean), Type 1-B. 7-Fluorobenzofurazan-4-sulfonamide 98% was acquired from Alfa Aesar (Kandel, Germany) whereas galantamine hydrobromide (purity > 98%) was obtained from TCI Chemicals (Tokyo, Japan).

Human proximal tubular epithelial cells (HK-2) and SH-SY5Y neuroblastoma cell lines were obtained from ATCC® (Rockville, MD, USA). Dulbecco’s modified Eagle medium nutrient mixture (DMEM/F12) culture medium, fetal bovine serum (FBS), PBS, l-glutamine, antibiotic solution (including penicillin and streptomycin), antibiotic–antimycotic solution (including penicillin, streptomycin and amphotericin B), and insulin-transferrin-selenium solution were purchased from Thermo Fisher (Grand Island, NY, USA); BDNF and cholesteryl ester (CE) 22:1 were obtained from Cymit Quimica (Spain); 3-(4,5-dimethylthiazol-2-yl)-2,5-diphenyltetrazolium bromide (MTT), NaCl, β-glycerophosphate, EDTA, n-octyl-β-d-glucopyranoside (BOG), protease inhibitor cocktail, NaF, sodium metavanadate (NaVO_4_), sodium pyrophosphate, LC–MS-grade isopropanol, ammonium formate, ammonium acetate, methyl tert-butyl ether (MTBE), toluene, β-carotene, and Val-Tyr-Val were obtained from Sigma-Aldrich (St Louis, MO, USA); lutein and zeaxanthin were acquired from Carbosynth Limited (Berkshire, UK); RA was purchased from Enzo Life Sciences GmbH (Germany); and amyloid-beta 1–42 (Aβ_1-42_) peptide was obtained from HelloBio (UK). LC–MS-grade acetonitrile (ACN) and LC–MS-grade methanol, ethanol, and urea were obtained from VWR Chemicals (Barcelona, Spain), whereas ultrapure water was obtained from a Millipore system (Billerica, MA, USA). Formic acid was purchased from Fisher Scientific (Waltham, MA, USA). The internal standard 12-[[(cyclohexylamino)-carbonyl]amino]-dodecanoic acid (CUDA) was purchased from LabClinics (Ann Arbor, MI, USA). The lipid standards lysophosphatidylcholine (LPC) 17:0, phosphatidylglycerol (PG) 17:0/17:0, ceramide (Cer) d18:1/17:0, monoacylglycerol (MG) 17:0/0:0/0:0, diacylglycerol (DG) 18:1/2:0/0:0, and triacylglycerol (TG) 17:0/17:1/17:0-d5 were provided by Avanti Polar Lipids (Alabaster, AL, USA). The isotope-labelled standards palmitic acid-d3, dl-alanine-3,3,3-d3, dl-glutamic acid-2,4,4-d3, d9-choline chloride, 15N2-l-arginine:HCl and l-methionine-d8 were obtained from Cambridge Isotope Laboratories Inc. (Andover, MA, USA).

### Carotenoid-enriched microalgal extract

*Dunaliella salina* (DS) extract was prepared in a semi-pilot Speed Helix supercritical fluid extractor (Applied Separations, Allentown, PA, USA), fixing the extraction conditions (pressure and temperature) that allowed the maximum extraction yield for this microalgae in a previous study [8]. Briefly, 25 g of *D. salina* powder was placed into a basket sandwiched between filter paper, within the high-pressure stainless steel extraction cell. Then, the CO_2_ was pumped into the extraction cell by a high-pressure pump until reaching the set values of pressure and temperature (400 bar and 45 °C, respectively). A constant flow rate (5 L min^−1^) of CO_2_ was set at the exit of the extraction cell, controlled using a CO_2_ gas flow meter, and maintained during 2 h. The resulting DS extract was collected in a 500-mL plastic bottle. The obtained dried extract was stored at − 20 °C until use.

### In vitro* bioactivity assays*

DS extract was evaluated using different in vitro assays related to AD, including anti-cholinergic activity inhibition of AChE and BChE, and anti-inflammatory and antioxidant activities (through LOX and ABTS assays, respectively), following the methods described in [13]. Galantamine, Trolox, and quercetin were used as positive controls for AChE (and BChE), ABTS, and LOX experiments, respectively. Each measurement was performed at least in triplicate and results were expressed as IC_50_ values (mean ± standard deviation), which indicate the concentration (μg/mL) of DS extract or positive control needed to reach the 50% of enzyme or radical inhibition capacity or compared to the control (without inhibitors).

### Cell culture and toxicity evaluation

HK-2 cells were cultured using the same protocol as in [13], together with an in vitro toxicity evaluation of the DS extract, with some modifications. In this case, a range of concentrations (from 2.5 to 40 µg/mL) of DS extract were added to the attached cells, and then, cells were incubated for 24 h. Ethanol was used to dissolve the extract and did not exceed the concentration of 0.4% (v/v) in cell medium. The viability of the cells was determined following the MTT assay method, also described in [13]. The toxicity of the extract is shown as relative cell viability, which is expressed as the percentage of living cells compared with control. All the experiments were performed at least in triplicate.

SH-SY5Y cells were grown, maintained, and differentiated as described by Medeiros et al. [9], with some modifications performed by our group [12]. In order to evaluate the in vitro toxicity of the DS extract in this cell line, the two highest non-toxic concentrations obtained for HK-2 cell line (10 and 20 µg/mL) were evaluated in SH-SY5Y cells. Cells were plated in 24-well plates at a density of 42,000 cells/cm^2^ for 24 h and, after cell attachment, the extract was added to the cells and incubated for 24 h. The viability of the cells was then determined by the MTT assay described above. Cell viability was expressed as a percentage of living cells compared to control (ethanol-treated). Again, ethanol did not exceed the concentration of 0.4% (v/v). All the experiments were performed in triplicate.

### Neuroprotection evaluation in SH-SY5Y cells

Two different experiments were performed to evaluate the neuroprotection capacity of the DS extract in SH-SY5Y cells: neuroprotection against Aβ_1-42_ and against l-glutamate, as in [14] with some modifications. Briefly, SH-SY5Y cells were plated as previously described in the “In vitro bioactivity assays” section, and after 24 h of cell attachment, cells were pre-treated with the highest non-toxic concentration of DS extract (20 µg/mL) for 24 h. Cell medium (DMEM/F12 supplemented with 1% FBS, 100 U/mL penicillin, 100 µg/mL streptomycin, 250 ng/mL antimycotic, and 0.4% of ethanol) was used as control. The next day, cells were incubated for another 24 h with cell medium (control), with Aβ_1-42_ (30 μM) or with l-glutamate (23 mM). The viability of the cells was finally determined by using the MTT assay as described above. All experiments were performed in triplicate.

### SH-SY5Y cell protein extraction and quantification

SH-SY5Y cells were seeded in P60 plates for 24 h followed by incubation with control medium (0.4% of ethanol) with or without 20 µg/mL of DS for 24 h (five plates per condition). The next day, the growth medium was removed by aspiration; cells were washed with PBS, trypsinized, and washed again with PBS. Then, cell pellets were centrifuged at 1,500 rpm for 7 min and proteins were extracted using 500 μL of the same lysis buffer as in [12, 15]. Samples were incubated for 60 min at 4 °C, sonicated for 30 min at 0 °C in ice-cold water bath, and then centrifuged at 10,000* g* at 4 °C for 15 min. The supernatants were collected, and protein concentration was quantified using Bradford assay (BioRad Laboratories, Hercules, CA).

### Intracellular metabolomics analysis of SH-SY5Y cells

#### Data acquisition

SH-SY5Y cells were seeded and treated following the same procedure as for the protein extraction (five P60 plates per condition), but a biphasic solvent extraction system consisting of cold methanol, MTBE, and water was used (as in [15]). Briefly, 225 μL of methanol at − 20 °C containing different internal standard compounds was added to the cell pellets (the list and concentration of each compound can be found in Electronic Supplementary Material (ESM Table S1) and vortexed for 30 s, and 750 μL of MTBE at − 20 °C containing CE (22:1) was added to the samples. Thereafter, the samples were ground using a Mixer Mill (Retsch MM301) for 2 min at 30 s^−1^, and 188 μL of LC–MS-grade water was added for phase separation. Finally, samples were vortexed and centrifuged for 2 min at 14,000 rpm, and 350 μL of the non-polar layer (upper fraction) and 125 μL of the polar layer (lower fraction) were collected from each sample and evaporated to dryness.

The dried non-polar samples were resuspended in 60 μL of methanol:toluene (9:1, v/v) mixture containing 50 ng/mL of CUDA for their analysis by CSH-Q-TOF MS/MS. Volumes of 3 μL (for ESI positive) and 5 μL (for ESI negative) were injected into a HPLC model 1290 (Agilent Technologies, Germany) coupled to a quadrupole Q-TOF series 6540 (Agilent Technologies, Germany) equipped with an Agilent Jet Stream thermal orthogonal ESI source. Agilent Mass Hunter Qualitative Analysis software (B.10.0) was used for MS control and data acquisition, and samples were analyzed using the same HPLC–MS conditions as previously described [12]. Method blanks and pooled mixtures of all control and DS-treated samples were included as quality control samples and were subjected to iterative MS/MS with mass error tolerance of 20 ppm and retention time (RT) exclusion tolerance of ± 0.2 min to increase the coverage of the MS/MS spectra acquired.

The dried polar samples were resuspended in 45 μL of ACN:water (4:1, v/v) with a mixture of internal standard compounds (shown in ESM Table S1) for their analysis by HILIC-Q-TOF MS/MS. Aliquots of 5 μL (for both ESI positive and ESI negative) were injected into the same LC–MS/MS instrument as mentioned above and compounds were separated using the same conditions as in [15]. The mass spectrometer was operated using the following parameters: capillary voltage, 3500 V; mass range, from 50 to 1700 m/*z*; nebulizer pressure, 35 psig; drying gas flow rate, 11 L/min and; dry gas temperature, 200 °C. The sheath gas flow was 11 L/min at 350 °C. MS/MS analyses were performed employing the auto MS/MS mode using 4 precursors per cycle, dynamic exclusion after two spectra (released after 0.2 min), and collision energies of 20 and 40 V. Mass accuracy was corrected using ions *m*/*z* 121.0509 (C_5_H_4_N_4_) and 922.0098 (C_18_H_18_O_6_N_3_P_3_F_24_) in ESI ( +) mode, and *m*/*z* 119.0363 (C_5_H_4_N_4_) and 966.0007 (C_18_H_18_O_6_N_3_P_3_F_24_ + formate) in ESI (-) mode. Quality control was performed in the same way as for the non-polar sample analyses, including iterative MS/MS to increase the coverage of the MS/MS spectra acquired.

#### Data processing

Data obtained from each analytical platform (CSH-Q-TOF MS/MS and HILIC-Q-TOF MS/MS) were processed separately by MS-DIAL software (v. 4.6) [16]. In-house *m*/*z* retention time libraries, the public LipidBlast MS/MS spectra library, and the NIST19 MS/MS database were used for metabolite annotation. The parameters used for processing were the same as in [12]. CUDA, CE (22:1), LPC (17:0), PG (17:0/17:0), Cer (d18:1/17:0), MG (17:0/0:0/0:0), DG (18:1/2:0/0:0), TG (17:0/17:1/17:0)-d5, and palmitic acid-d3 internal standard compounds were used for retention time correction in CSH-Q-TOF MS/MS data; and CUDA, dl-alanine-d_3_, dl-glutamic acid-d_3_, choline-d_9_, 15N_2_-l-arginine, l-methionine-d_8_, and Val-Tyr-Val internal standard compounds were used for retention time correction in HILIC-Q-TOF MS/MS data. Metabolites were annotated following the Metabolomics Standard Initiative (MSI) guidelines [17] using the same terminology as in [12, 15].

#### Statistical analysis

The lists of metabolites obtained from each analytical platform and ionization modes were analyzed independently. In all cases, unknown metabolites, metabolites with a maximum peak height below three times the average height in the blank samples, and metabolites with a maximum height below 1000 units were removed. Metabolites present in more than three samples for at least one group were retained. Thereafter, missing values were imputed by half of the minimum value, and data were processed using MS-FLO tool [18]. By using this software, duplicated metabolites and isotopes were detected and manually filtered, and the height of the different adducts from the same compound was combined. The set of metabolites was then normalized by using the sum peak height of all identified metabolites in the analyses (mTIC). Principal component analysis (PCA) and partial least square-discriminant analysis (PLS-DA) were performed by using MetaboAnalyst 5.0 web-based software [19], with previous “Auto scale” normalization. PLS-DA models were evaluated according to the cross-validation of *R*^2^ and *Q*^2^, and exported variable importance in projection (VIP) scores was obtained. Finally, the non-parametric Mann–Whitney *U* test with false discovery rate (FDR) correction was performed, setting a fold change (FC) cutoff of 0.67 > FC > 1.5, and a FDR of 0.05 for considering metabolites as significantly altered. Chemical similarity enrichment analysis was performed using the ChemRICH bioinformatic tool [20]. Data matrices from each platform and ionization mode were combined to generate a joint dataset and data with the highest retention time similarity (from the respective *m*/*z* retention time libraries), highest MS/MS similarity score, highest peak intensity, and/or better peak shape were retained. Thereafter, the web-based Chemical Translation Service (http://cts.fiehnlab.ucdavis.edu/batch) was used to obtain PubChem Compound Identifiers (CID) from the InChiKey or compound names, and the simplified molecular-input line-entry system (SMILES) codes were obtained from the MSP files or from the PubChem Compound Identifier Exchange service (https://pubchem.ncbi.nlm.nih.gov/idexchange/idexchange.cgi).

### Carotenoid analysis of SH-SY5Y cell culture medium

In addition to the intracellular metabolomic analyses, the carotenoid composition of SH-SY5Y cell culture medium was determined after the addition of DS extract at 20 μg/mL (*t* = 0 and 24 h), using cell culture medium without cells as control samples. Aliquots of 1 mL of each sample were collected and dried, and carotenoids were extracted by using the same biphasic extraction method as in the “Intracellular metabolomics analysis of SH-SY5Y cells” section, with the exception of the addition of standard compounds. The non-polar layers were collected, evaporated to dryness, and resuspended in HPLC-grade methanol to a final concentration of 1.0 mg/mL. Aliquots of 10 μL were injected into the same LC–MS/MS instrument as mentioned above, but using a diode-array detector (DAD) and an atmospheric pressure chemical ionization interface. Compounds were separated using a YMC-C30 reversed-phase column (250 × 4.6 mm, 5 μm particle size; YMC Europe, Schermbeck, Germany), using a YMC-C30 pre-column (10 × 4 mm, 5 μm particle size). The separation conditions were the same as previously described by Bueno et al. [8]. The mass spectrometer was operated in positive mode using the following parameters: capillary voltage, 3500 V; mass range, from 50 to 1100 m/*z*; nebulizer pressure, 40 psig; drying gas flow rate, 8 L/min; drying gas temperature, 300 °C; vaporizer gas temperature, 350 °C; skimmer voltage, 45 V; fragmentor voltage, 110 V. MS/MS analyses were performed employing the auto MS/MS mode using 4 precursors per cycle, dynamic exclusion after three spectra (released after 0.2 min), and collision energies of 20 and 40 V. Mass accuracy was corrected using ions *m*/*z* 121.0509 (C_5_H_4_N_4_) and 922.0098 (C_18_H_18_O_6_N_3_P_3_F_24_). DAD signals were acquired at 240–640 nm. Commercial standards (β-carotene, zeaxanthin, and lutein), UV absorption spectrum, MS/MS spectrum, retention times, and/or elution order reported in the literature were used for carotenoid identification. The area under curve (AUC) obtained at 450 nm for each identified carotenoid was compared among samples and ANOVA with Tukey’s post hoc test was employed to determine any significant differences between mean values using *p*-value < 0.05.

## Results

### In vitro* neuroprotective potential assay of DS extract*

The potential neuroprotective effect of DS extract was evaluated through multiple in vitro assays, including the study of its inhibitory capacity against AChE, BChE, and LOX enzymes, together with its antioxidant activity. To compare the obtained values, positive controls were also included in all cases. Thus, the IC_50_ values for each experiment are included in Table 1. Regarding the results for anti-AChE and BChE assays, IC_50_ values of DS extract are far from those for galantamine, a commercial pharmaceutical drug developed to block those activities and used as control. However, according to the general classification of efficacy for natural extracts [21], the DS extract can be included into the moderate potency AChE inhibitors capacity group (20 < IC_50_ < 200 μg/mL).

**Table 1:** IC_50_ values ± SD (μg/mL) for AChE, BChE, LOX, and ABTS assays obtained for *Dunaliella salina* extract (*n* = 3) and positive controls (galantamine^a^, quercetin^b^, and Trolox^c^) (*n* = 3)

Sample	IC_50_ (µg/mL)			
	**AChE**	**BChE**	**LOX**	**ABTS**
Control	0.40 ± 0.02^a^	2.45 ± 0.02^a^	19.71 ± 0.24^b^	2.5 ± 0.02^c^
DS extract	73.14 ± 3.46	96.56 ± 6.58	35.84 ± 1.43	12.41 ± 0.11

*AChE*, acetylcholinesterase; *BChE*, butyrylcholinesterase; *LOX*, lipoxygenase

The results of the anti-inflammatory potential of DS extract (LOX) showed a good inhibitory capacity since the LOX IC_50_ value was only 1.8-fold increased with respect to the positive control (quercetin). Finally, ABTS assay results indicated that the DS extract exhibited lower antioxidant capacity in comparison to the control used (Trolox), although the value obtained can be considered as low (12.41 µg/mL).

### Toxicity evaluation of DS extract in HK-2 and SH-SY5Y cell models

The in vitro toxicity evaluation of the DS extract was firstly carried out on HK-2 cells in order to screen a wide range of concentrations (from 2.5 to 40 µg/mL). The highest concentration (40 µg/mL) of DS extract significantly affected the cell viability in comparison to control, decreasing up to 13.7% of living cells (see ESM, Fig. S1). The rest of the concentrations tested, from 2.5 to 20 µg/mL, did not show any statistically significant decrease in cell viability. Consequently, an in vitro toxicity evaluation on SH-SY5Y cells was performed using the two highest non-toxic concentrations determined for HK-2 cells (10 and 20 µg/mL). The results showed that none of these concentrations altered the cell viability in comparison to controls, and therefore, 20 µg/mL was the DS extract concentration selected to pre-treat SH-SY5Y cells and to evaluate its potential as a neuroprotective agent.

### *Neuroprotective activity of DS extract against Aβ*_*1-42*_* and l-glutamate in SH-SY5Y cells*

According to the above-mentioned toxicity results, differentiated SH-SY5Y cells were seeded and incubated with 20 µg/mL of DS extract for 24 h. After that, the neurotoxic agent Aβ_1-42_ or l-glutamate was added for another 24 h at a concentration of 30 µM or 23 mM, respectively. Controls containing only cell growth medium were also included to determine the maximum cell viability.

**Fig. 1:**
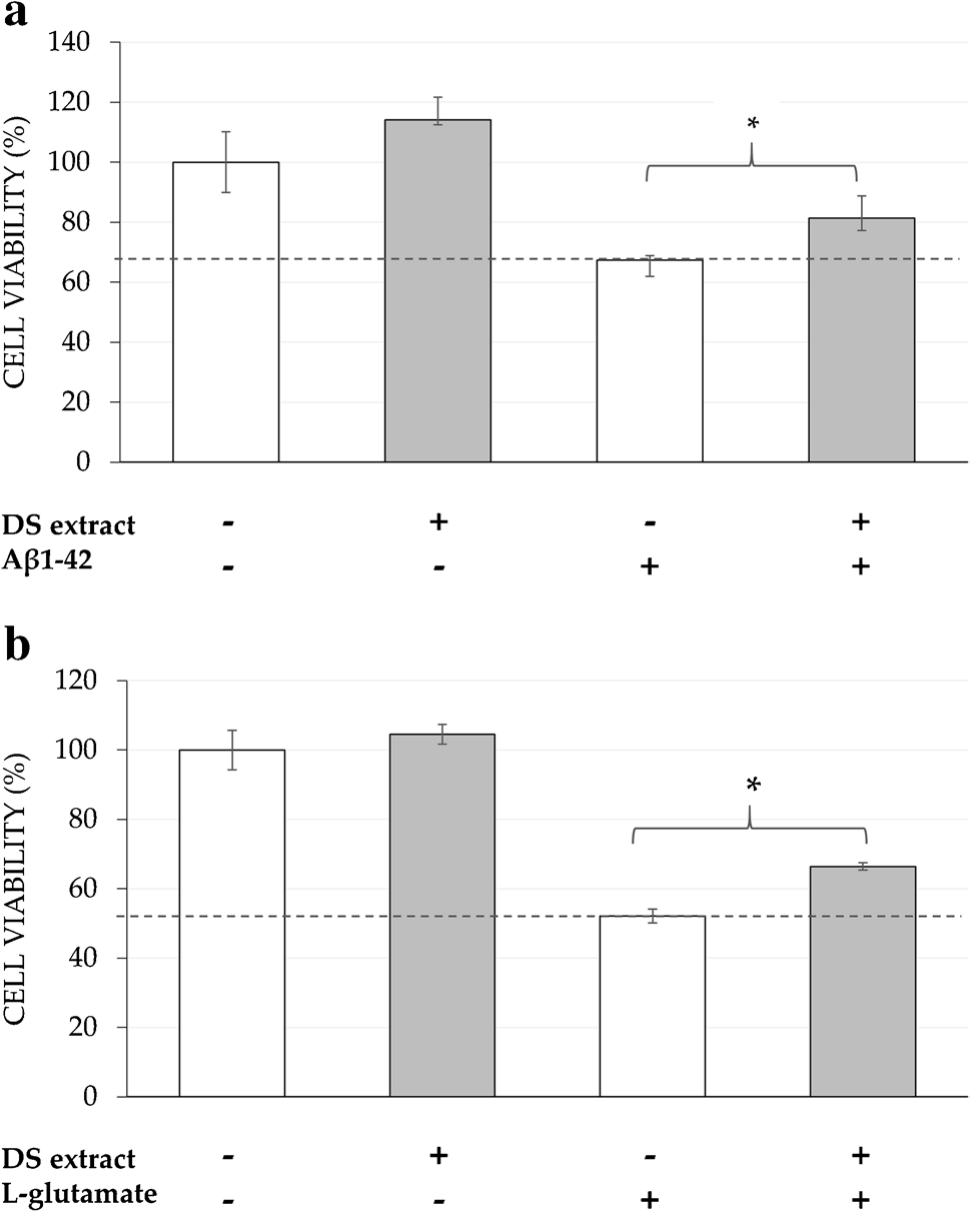
Neuroprotective effect of pre-incubation of *D. salina* (DS) extract against the neurotoxic agents (a) Aβ_1-42_ (30 µM) and (b) l-glutamate (23 mM) in differentiated SH-SY5Y cells. Non Aβ_1-42_-treated cells were used as control, together with only DS extract-treated cells at 20 μg/mL. The results are mean (*n* = 3) ± SD. * denotes statistical differences between control and DS extract when neurotoxic agent is added (*p < 0.05)

As it can be observed in Fig. 1A, in the absence of DS extract, the neurotoxic agent Aβ_1-42_ at 30 µM reduced cell viability to 67% in comparison to the control (non-treated). When DS extract was added as a pre-treatment before Aβ_1-42_ induction, a significant difference of the cell viability could be observed (reaching up to 81% of cell viability) in comparison to Aβ_1-42_-induced cells (67%).

Similarly, when the neurotoxic agent l-glutamate (at 23 mM) was added to cells, cell viability decreased to 58% compared to control cells (non-treated) (Fig. 1B). Moreover, when DS extract was added as a pre-treatment, again cell viability achieved a significant increase (66% of cell viability) in comparison to l-glutamate-induced cells. It can also be observed that DS extract pre-treatment alone maintained the cell viability at maximum.

### Protein content

Before studying the metabolomics changes produced by DS extract treatment on the SH-SY5Y cells, a protein content assay was performed to complement the toxicity/viability assay results. In this sense, five P60 cultured plates were treated for 24 h with 20 µg/mL of DS extract (diluted in 0.4% v/v ethanol) and five plates were seeded as control (only ethanol-treated). In total, 37.3 ± 2.9 µg of proteins could be obtained from the control and 40.2 ± 2.5 µg from extract-treated cells, indicating no differences in the protein content after the treatment (*p*-value = 0.126 after two-sample *t*-test).

### Metabolomics identification in SH-SY5Y intracellular medium

#### Lipids

**Fig. 2:**
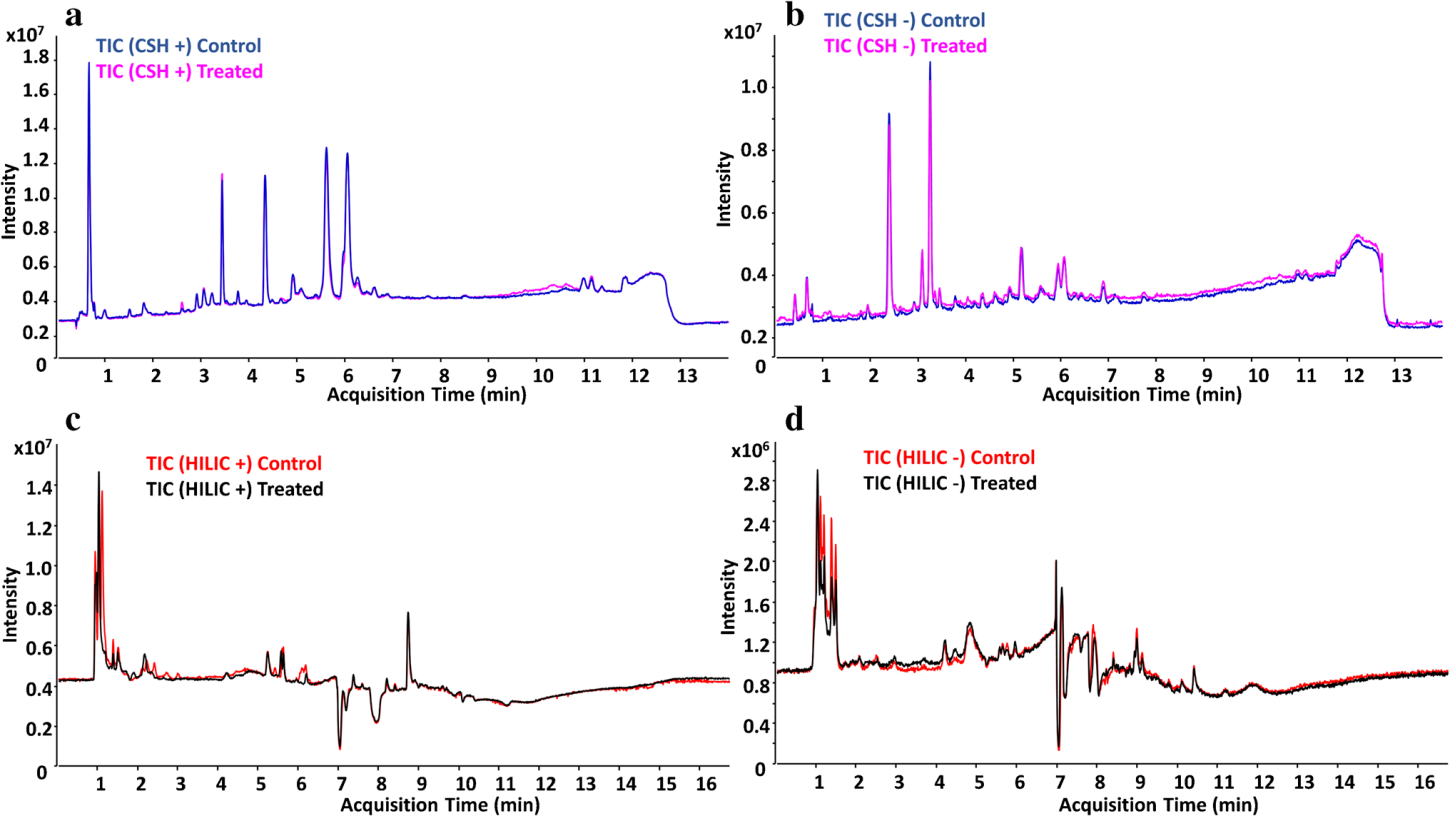
Representative total ion current (TIC) chromatogram of the lipid profile in ESI ( +) (**a**) and ESI (-) (**b**), and the polar fraction in ESI ( +) (**c**) and ESI (-) (**d**), for both treatment and control groups

In order to broaden the coverage of identified non-polar compounds, an untargeted lipidomics analysis on SH-SY5Y cells was performed based on CSH-Q-TOF MS/MS analysis and using two complementary ionization modes (ESI ( +) and ESI (-)). A representative TIC of the lipid profile in both treatment and control groups is shown in Fig. 2A and B. The relative standard deviation of the internal standards included during sample preparation is shown in ESM, Table S2. After data post-processing, the CSH-Q-TOF MS/MS analysis resulted in the annotation of 246 lipids: 135 in ESI ( +), 49 in ESI (-), and 62 ionizing in both ionization modes (ESM, Table S3). For those lipids annotated in both ionization modes, good Pearson’s correlation *r* values between the RT and FC values (*r* = 0.992 for RT; *r* = 0.992 for FC) were obtained (ESM, Fig. S2). Identified lipids were classified in more than 15 lipid classes, including ceramides (Cer), fatty acids (FA), lysophosphatidylcholines (LPC), phosphatidylcholines (PC), ether PC, phosphatidylethanolamines (PE), PE ethers, phosphatidylglycerols (PG), phosphatidylinositols (PI), phosphatidylserines (PS), sphingomyelins (SM), diglycerides (DG), triglycerides (TG), and ether TG, among others.

#### Polar compounds

The polar fraction obtained from the DS extract-treated cells was analyzed using HILIC-TOF MS/MS, also using ESI ( +) and ESI (-) ionization modes (a representative TIC of the treatment and control groups is shown in Fig. 2C and D). Data processing of each ionization mode was performed independently, and the relative standard deviations of the internal standards included for this analysis are shown in ESM, Table S2. The HILIC-Q-TOF MS/MS analysis allowed the annotation of 68 compounds: 38 in ESI ( +), 16 in ESI (-), and 14 in both ionization modes (ESM, Table S4). Thus, most remarkable polar metabolites found in the samples were distributed in amino acids, carnitines, deoxyadenosines, diamines, dipeptides, glutamates, guanidines, or hexoses. For the compounds annotated in both ESI ( +) and ESI (-), Pearson’s correlation *r* values between the RT and FC values were as follows: *r* = 0.999 for RT and *r* = 0.924 for FC, as it can be found in ESM, Fig. S2.

#### Multivariate and univariate analyses

PCA and PLS-DA data from the non-polar and polar fractions were analyzed separately. Regarding the lipidic fraction, the PCA of data from both ionization modes indicated that the two groups of samples (DS-treated and control) are clearly different (ESM, Fig. S3, A and B). Also, the PLS-DA data for the two groups of metabolites indicated that the percentage of variation explained and the predictive ability of the models based on the first two components is good (*R*^2^ = 0.999 and *Q*^2^ = 0.989 for ESI ( +); *R*^2^ = 0.999 and *Q*^2^ = 0.989 for ESI (-)) (ESM, Fig. S3, C and D). Furthermore, the VIP scores indicated that PCs (36:3 A, 36:4; A, 38:6 A, 34:4, 34:3 B, and 38:5 B) and TGs (54:5, 58:7, and 56:7 A) are the most relevant lipids for the separation of the DS-treated vs control cells in ESI ( +), whereas PCs (34:4, 34:4, 36:4 A, 36:5 B, 38:5 B, 38:6, and 36:3) are the most important compounds for group separation in ESI (-) (ESM, Table S3).

Complementary to these results, the Mann–Whitney univariate analysis showed a total of 44 and 6 lipids significantly altered in ESI ( +) and ESI (-), respectively. More specifically, 35 lipids were significantly increased in ESI ( +), including PCs (36:5 A, 36:4 A, 34:3 B, 36:4 B, among others) and TGs (56:8 A, 52:5, 54:7 B, 56:10, or 54:6 B, as some examples), while 3 lipids were decreased (PC 40:7 B; TG 56:2, and TG 55:2). In ESI (-), 9 lipids were increased, including FAs (18:3, 20:3, 20:4, 20:5), PCs (35:4, 36:5 B), PE 36:3, PE O-36:6, and PI 38:3; and 3 PGs were decreased (44:12, 40:7, and 36:2). The whole list of increased and decreased lipids is shown in Table 2 and Table 3, respectively.

**Table 2:** Increased lipids in SH-SY5Y cells after the treatment with *Dunaliella salina* extract at 20 μg/mL (*n* = 5) in comparison to control conditions (*n* = 5) after 24 h. Significance is determined using a FC threshold ≥ 1.5 and the Mann–Whitney *U* test with FDR < 0.05

Lipid name	Ionization mode	MSI level	FC	*p*-value	FDR
TG 58:6	ESI ( +)	1	1.5317	7.94E-03	0.014086
TG 52:3	ESI ( +)	1	1.5887	7.94E-03	0.014086
TG 56:8; B	ESI ( +)	2b	1.6453	7.94E-03	0.014086
TG 50:3; A	ESI ( +)	2b	1.6827	7.94E-03	0.014086
TG 54:5; B	ESI ( +)	2b	1.8438	7.94E-03	0.014086
TG 50:3; B	ESI ( +)	1	2.0165	7.94E-03	0.014086
PE 36:3	ESI (-)	2b	2.2474	7.94E-03	0.016941
PI 38:3|PI 18:0_20:3	ESI (-)	2a	2.289	7.94E-03	0.016941
PC 35:3	ESI ( +)	2b	2.3779	7.94E-03	0.014086
TG 53:4	ESI ( +)	2b	2.4063	7.94E-03	0.014086
TG 54:4	ESI ( +)	1	2.5237	7.94E-03	0.014086
PC 36:3; A	ESI ( +)	2b	2.6273	7.94E-03	0.014086
TG 56:6	ESI ( +)	1	2.7066	7.94E-03	0.014086
PC 38:5; B	ESI ( +)	2b	2.7628	7.94E-03	0.014086
PE O-36:6 | PE O-16:1_20:5	ESI (-)	2a	3.0815	7.94E-03	0.016941
TG 58:7 | TG 18:0_18:2_22:5	ESI ( +)	2a	3.106	7.94E-03	0.014086
FA 20:3	ESI (-)	2b	3.1968	7.94E-03	0.016941
TG 58:9	ESI ( +)	2b	3.23	7.94E-03	0.014086
FA 18:3	ESI (-)	2b	3.258	7.94E-03	0.016941
PC O-38:7	ESI ( +)	2a	3.2866	7.94E-03	0.014086
FA 20:5	ESI (-)	2b	3.6994	7.94E-03	0.016941
PC 36:5; D	ESI ( +)	2b	4.3572	7.94E-03	0.014086
PC 36:5; B	ESI (-)	2b	4.6587	7.94E-03	0.016941
PC 34:4	ESI ( +)	2b	5.0593	7.94E-03	0.014086
PC 38:6; A	ESI ( +)	2b	5.3763	7.94E-03	0.014086
TG 53:5	ESI ( +)	2b	5.6114	7.94E-03	0.014086
PC p-36:5/PC o-36:6	ESI ( +)	2b	5.6156	7.94E-03	0.014086
TG 56:7; A	ESI ( +)	2b	7.0426	7.94E-03	0.014086
PC 36:4; B	ESI ( +)	2b	7.2773	7.94E-03	0.014086
TG 54:6; C	ESI ( +)	2b	7.7059	7.94E-03	0.014086
TG 52:4	ESI ( +)	1	8.8299	7.94E-03	0.014086
PC 35:4	ESI (-)	2b	9.2395	7.94E-03	0.016941
FA 20:4	ESI (-)	2b	9.9286	7.94E-03	0.016941
TG 52:6	ESI ( +)	2b	11.788	7.94E-03	0.014086
TG 54:5 | TG 18:1_18:1_18:3	ESI ( +)	2a	12.212	7.94E-03	0.014086
TG 58:10	ESI ( +)	2b	13.217	7.94E-03	0.014086
PC 34:3; B	ESI ( +)	2b	14.584	7.94E-03	0.014086
TG 54:6; B	ESI ( +)	1	16.637	7.94E-03	0.014086
TG 56:10	ESI ( +)	2b	18.493	7.94E-03	0.014086
TG 54:7; B	ESI ( +)	2b	20.105	7.94E-03	0.014086
TG 52:5	ESI ( +)	1	22.05	7.94E-03	0.014086
TG 56:8; A	ESI ( +)	2b	22.978	7.94E-03	0.014086
PC 36:4; A	ESI ( +)	1	34.748	7.94E-03	0.014086
PC 36:5; A	ESI ( +)	2b	235.33	7.94E-03	0.014086

*FA*, fatty acids; *PC*, phosphatidylcholine; *PE*, phosphatidylethanolamines; *PI*, phosphatidylinositol; *TG*, triglyceride; *FC*, fold change; *FDR*, false discovery rate; *MSI*, Metabolomics Standards Initiative; *RT*, retention time

MSI level 1: *m*/*z*, MS/MS, RT; MSI level 2a: *m*/*z*, MS/MS; MSI level 2b: *m*/*z*, RT

**Table 3:** Decreased lipids in SH-SY5Y cells after the treatment with *Dunaliella salina* extract at 20 μg/mL (*n* = 5) in comparison to control conditions (*n* = 5) for 24 h. Significance is determined using a FC threshold ≤ 0.67 and the Mann–Whitney *U* test with FDR < 0.05

Lipid name	Ionization mode	MSI level	FC	*p*-value	FDR
PG 44:12|PG 22:6_22:6	ESI (-)	2a	0.47009	0.0079365	0.016941
PG 40:7|PG 18:1_22:6	ESI (-)	2a	0.50576	0.0079365	0.016941
PG 36:2|PG 18:1_18:1	ESI (-)	2a	0.53546	0.0079365	0.016941
PC 40:7; B	ESI ( +)	2b	0.629	0.0079365	0.014086
TG 56:2	ESI ( +)	2b	0.63651	0.0079365	0.014086
TG 55:2	ESI ( +)	2b	0.64838	0.0079365	0.014086

*PC*, phosphatidylcholine; *PG*, phosphatidylglycerol; *TG*, triacylglycerol; *FC*, fold change; *FDR*, false discovery rate; *MSI*, Metabolomics Standards Initiative; *RT*, retention time

MSI level 2a: *m*/*z*, MS/MS; MSI level 2b: *m*/*z*, RT

On the other hand, the PCA of the polar compounds showed no differences between the two groups of samples (ESM, Fig. S4A and B); and the PLS-DA data obtained for this fraction indicated that the separation between both groups is not as good as for the lipid compounds (*R*^2^ = 0.860 and *Q*^2^ = 0.054 for ESI ( +); *R*^2^ = 0.815 and *Q*^2^ =  − 0.814 for ESI (-)) (ESM, Fig. S4, C and D). This analysis only suggested pantothenic acid as a relevant compound for the separation of both groups (VIP = 2.235 and 2.386 in ESI ( +) and ESI (-), respectively; ESM, Table S4), but the Mann–Whitney showed no significantly altered compounds after the treatment with the DS extract.

#### Global metabolomics analysis

**Fig. 3:**
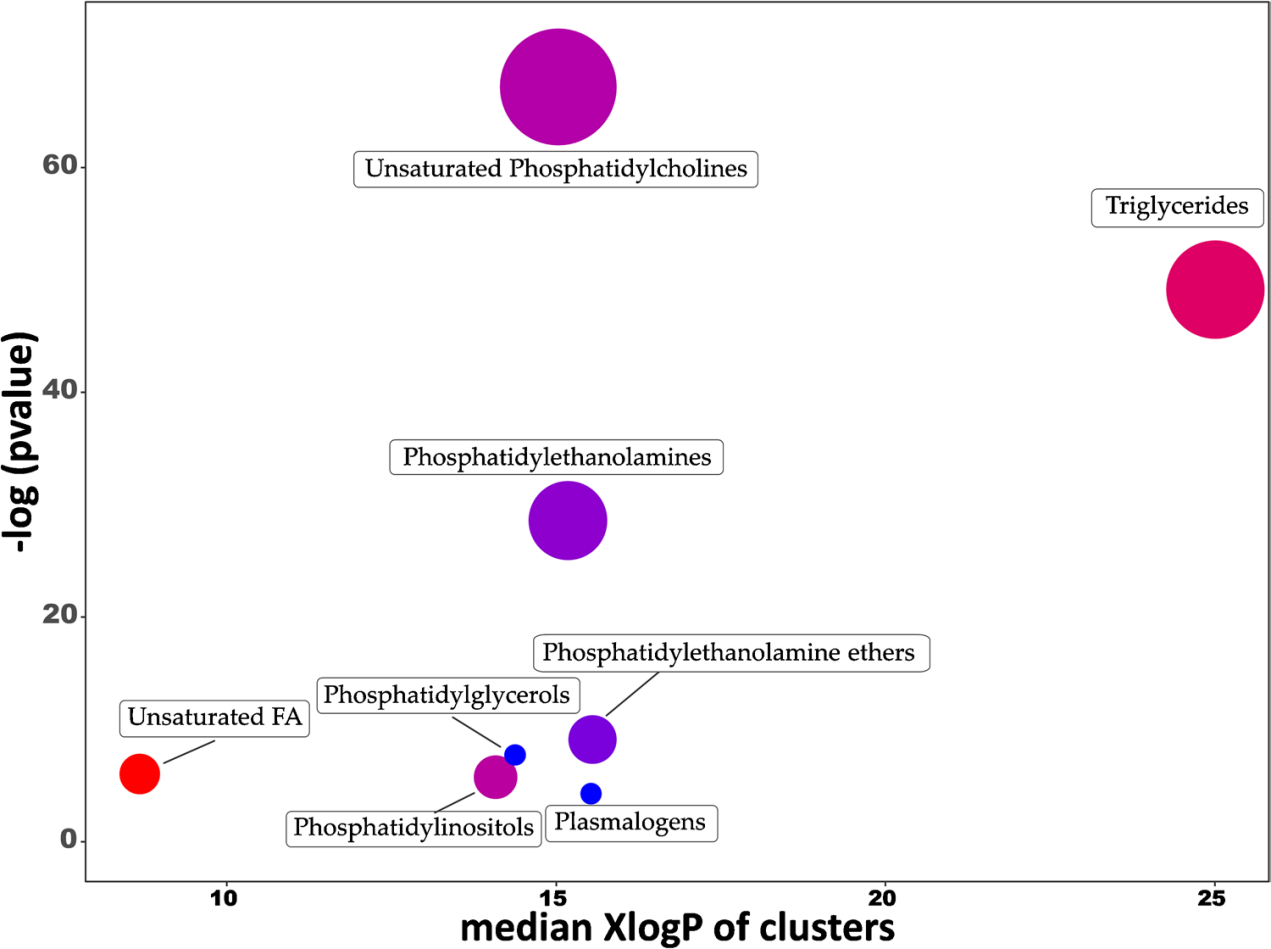
Chemical similarity enrichment analysis of metabolomics data from SH-SY5Y cells treated with *D. salina* (DS) extract at 20 μg/mL (*n* = 5) compared to control conditions (0.4% ethanol v/v) for 24 h (*n* = 5). Statistical enrichment analysis utilized chemical similarity and ontology mapping to generate metabolite clusters. The *y*-axis shows most significantly altered clusters on top; *x*-axis shows XlogP values of lipid clusters. Cluster colors give the proportion of increased or decreased compounds (red = increased, blue = decreased) in each cluster. Chemical enrichment statistics is calculated by Kolmogorov–Smirnov test. Only enrichment clusters are shown that are significantly different at *p* < 0.05. Plots and calculations were done using ChemRICH

ChemRICH tool was used to obtain a global metabolomics view in which significantly altered chemical compound classes after the treatment can be easily recognized (Fig. 3). Unsaturated PC was the most altered cluster (with 35 compounds decreased and 20 increased), followed by TG class, with most of the compounds significantly increased. PEs, PE ethers, PGs, unsaturated FA classes, and plasmalogens were also significantly altered (mostly decreased), and only a few PIs were significantly altered with some increased, others decreased. The complete list of chemical classes and their *p*-values can be found in ESM, Table S5.

### Carotenoid composition in SH-SY5Y extracellular medium

The differences in SH-SY5Y extracellular medium after the addition of DS extract were analyzed in terms of carotenoid composition. To better understand the results, four conditions were tested in P60 plates, including (a) cell-seeded plates + DS extract at *t* = 0 h; (b) cell-seeded plates + DS extract at *t* = 24 h; (c) non-cell-seeded plates + DS extract at *t* = 0 h; and (d) non-cell-seeded plates + DS extract at *t* = 24 h. Through HPLC–DAD-MS/MS analysis, lutein, zeaxanthin, two cryptoxanthins, all*-tran*s-β-carotene, and 9-*cis*-β-carotene isomer were identified. Carotenoid abundance is presented in Table 4 as the value of the AUC, obtained from HPLC–DAD data analysis. The ratios 24 h/0 h (for cell-seeded and non-cell-seeded conditions) are also given, which allow a rapid overview of increasing (ratio > 1) or decreasing (ratio < 1) signals over time.

**Table 4:** Evaluation of the presence and absence of SH-SY5Y cells on identified carotenoids in cell medium before and after the treatment with *Dunaliella salina* extract at 20 μg/mL for 24 h (*n* = 5). Values are expressed as the AUC obtained by peak integration of DAD absorption spectrum of HPLC–DAD-MS/MS analysis. Different letters in same row indicate significant differences between samples after ANOVA with Tukey’s post hoc, *p*-value < 0.05

Carotenoid	Cells		Non-cells		Ratio 24 h/0 h		
	**0 h**	**24 h**	**0 h**	**24 h**	**Cells**	**Non-cells**	***t*-test**
Lutein	93 ± 5^a^	54 ± 8^b^	89 ± 16^a^	49 ± 7^b^	0.583	0.556	0.614
Zeaxanthin	40 ± 2^a^	16 ± 2^b^	40 ± 7^a^	15 ± 3^b^	0.394	0.386	0.848
Cryptoxanthin-type	68 ± 2^b^	121 ± 15^a^	68 ± 11^b^	122 ± 16^a^	1.786	1.805	0.899
Cryptoxanthin-type	92 ± 6^a^	108 ± 14^a^	79 ± 24^a^	107 ± 19^a^	1.171	1.347	0.206
all-*trans*-β-Carotene	513 ± 44^a^	246 ± 38^b^	637 ± 160^a^	279 ± 32^b^	0.479	0.437	0.335
9-*cis*-β-Carotene isomer	3787 ± 183^a^	1468 ± 155^b^	3557 ± 613^a^	1405 ± 211^b^	0.388	0.395	0.826

*AUC*, area under curve

Results indicated that all identified carotenoids were significantly altered in cell-seeded and non-cell-seeded conditions after 24 h, with the exception of cryptoxanthin (II). Specifically, lutein, zeaxanthin, and all-*trans*-β-carotene were found decreased, while cryptoxanthin (I) was found significantly increased. In addition, no significant differences in the 24 h/0 h ratio between cells and non-cells were observed, indicating that the differences in the cell culture medium are cell-independent and suggesting that these carotenoids are not being metabolized or incorporated by the cells.

## Discussion

AD is a multifactorial pathophysiology characterized by neuroinflammation, extensive oxidative damage, synaptic loss, and neuronal cell death. In this sense, the design of multi-target strategies to prevent or delay its progression is a challenging goal. AD has been associated with a decrease of AChE levels in the brain that leads to an abnormal cholinergic neurotransmission, affecting several brain functions, attention, and memory impairment [22]. The inhibition of chlorinesterase (ChE) enzymes, which are responsible for acetylcholine hydrolysis, could help to delay the onset of AD. Up to now, only a few ChE inhibitors (specifically donepezil, galantamine, and rivastigmine) have been considered a relatively successful AD treatment [23], although other natural compounds, such as carotenoids, have been recently reported as potential neuroprotective agents [24].

In this regard, a potential carotenoid-enriched extract obtained from *D. salina* microalgae was evaluated through several in vitro neuroprotective activities, including the inhibition of cholinesterase enzymes (AChE and BChE), together with its potential as anti-inflammatory (determined by LOX assay) and antioxidant (determined by ABTS assay) agents. Results indicated that DS extract presents moderate potency as AChE inhibitor, which is a promising result compared to other extracts from natural matrices [13, 21]. Indeed, in terms of antioxidant activity, the IC_50_ value obtained for DS extract is lower than that for other potential sources such as rosemary or olive leaves’ extracts [13]. This fact could be associated with its high composition in carotenoids, specifically β-carotene, lutein, or zeaxanthin, whose implication as natural antioxidant and anti-neuroinflammation agents in neurodegenerative diseases has been already demonstrated [24]. Moreover, recent studies have confirmed that β-carotene, the main carotenoid in DS extract, has a protector effect against neuroinflammation through the reduction of oxidative stress, oxidative stress-induced damage, and the increase of motor functions [25].

Besides the oxidative stress and the neuroinflammation, other major factors contributing to the progression of AD are the accumulation of NFT and Aβ plaques, or through the excitotoxic pathway, which is defined as a toxic reaction caused by an over-accumulation of amino acids. For instance, the overexposure of neurons to the amino acid glutamate leads to neuronal damage and apoptosis [26]. In this regard, the neuroblastoma SH-SY5Y cell model has been widely used as an in vitro model for AD researches [9] such as the study of the Aβ_1-42_ inhibitory activity of different natural components [13]. The present study assessed the potential neuroprotective effect of DS extract pre-treatment in Aβ and l-glutamate-induced SH-SY5Y cells. An in vitro toxicity assay of DS extract was carried out before the neuroprotection experiments and 20 µg/mL was selected as working concentration since it did not alter the cell viability of any of the cell lines tested, compared to control cells. Thus, the results indicated that the pre-treatment with 20 µg/mL of DS extract was able to partially attenuate cell death caused by these neurotoxic agents (30 µM of Aβ and 23 mM of l-glutamate), although it was not enough to reach the maximum neuroprotection.

Similarly, other studies have confirmed the neuroprotective effect of carotenoids in Aβ-induced cell lines. As an example, lutein, whose presence has been confirmed in DS extract, has been described as a protective agent against Aβ_25-35_ peptide-induced oxidative stress and apoptosis in cerebrovascular endothelial cells b.END.3 [27]. Other carotenoids were confirmed to exhibit an in vitro inhibitory effect on Aβ-induced apoptosis in SH-SY5Y cells, or even exerting a protective effect on amyloid fibril formation of Aβ peptide by avoiding Aβ assembly through hydrogen bonding and van der Waals interactions [28, 29].

With regard to glutamate-induced excitotoxicity in SH-SY5Y cell, natural compounds such as pentacyclic triterpenes or edible seaweed extracts have been described to exhibit strong neuroprotective activity [30]. However, the role of carotenoid excitotoxicity has not been deeply explored. To the best of our knowledge, only a recent study has evaluated the effect of astaxanthin, a potent antioxidant carotenoid, in glutamate-induced SH-SY5Y. The results suggested that this carotenoid could prevent mitochondrial impairment, and thus exert a neuroprotective effect [31].

In parallel, a comprehensive metabolomics study, using CSH-Q-TOF MS/MS, HILIC-Q-TOF MS/MS, and advanced bioinformatics tools, was performed to understand the possible neuroprotective mechanism exerted by the pre-treatment of DS extract on SH-SY5Y cells. In this regard, a total of 246 lipids and 38 polar compounds were annotated in the intracellular cell extracts. Among them, only lipidic compounds were found significantly altered in DS extract-treated cells in comparison to non-treated cells, possibly due to the non-polar nature of the extract or due to the lower sensitivity and reproducibility of the HILIC separation system used.

The brain is enriched in lipids (up to 50% of dry brain weight), which are the basic structural component of neuronal cell membrane and play a relevant role in human brain function. The study of cerebral lipids (including phospholipids, glycolipids, cholesterol, among others) has become an interesting topic of investigation within neurodegenerative diseases, since they have been related to alterations of the lipid homeostasis [32]. In line with this, our lipidomics results show that, in general terms, three groups of lipids are significantly increased after the treatment with DS extract (PCs, TGs, and FAs), whereas one group was found to be decreased (PGs).

Regarding PC group, the main lipids whose abundance was increased after the treatment were PC 36:5 A, 36:5 A, 34:3 B, and 35:4. Among other phospholipids, PC have essential functional and structural roles in the integrity and functionality of cell membranes, and their metabolism has been associated with several key molecular pathways intrinsic to AD [33]. In this sense, an interesting study was carried out by Kim et al. [34], who found that two specific PCs (PC 36:5 and PC 38:6) were found to be depleted in plasma from AD patients compared to the control group [34]. A similar study showed that other PCs (36:6, 38:0, 38:6, 40:1, 40:2, and 40:6) were found decreased in plasma from AD patients [35]. Furthermore, the neuroprotective effect of PC on cultured neurons has been already studied by Ko et al. [36], which showed that neurons pre-treated with PC were protected against a later exposition to Aβ_1-42_ [36]. This could explain that the increased values of some PCs observed after DS extract cell pre-treatment could be associated with the relative neuroprotective effect observed.

TG group was also significantly increased after the DS extract treatment, being TG 56:8 A, 52:5, 54:7 B, 56:10 and 54:6 B the most outstanding lipids. TGs are not very abundant in the brain, and there is not a clear relationship between TG and AD in the literature. For example, it has been reported that TGs were generally increased in post-mortem cerebrospinal fluid of patients with mild cognitive impairment and AD, although these changes were not significant [37]. In another study in AD transgenic mice, an increase of several TGs were observed in plasma, compared to controls [38]. However, Tajima et al. [39] found that the levels of most TGs studied (TG 52:3, 53:6, and 54:4) were decreased in brain tissues and plasma obtained from mice expressing mutated human amyloid precursor protein and tau protein compared to wild-type mice [39].

Another group of compounds whose abundance was significantly increased after extract treatment is FA. Specifically, the polyunsaturated FAs (PUFAs) 20:4, 20:5, 18:3, and 20:3 were found to be from 3- to tenfold increased. PUFAs are known to be involved in brain development and cognitive function. Once PUFAs are released from the cell membrane, they can participate in signal transduction, regulating several processes within the brain, such as neurotransmission, cell survival, or neuroinflammation [40]. Interestingly, different studies have demonstrated that some PUFAs (such as FAs 20:4, 20:5 and 18:3) are known to exhibit beneficial neuroprotective effects in AD, preventing Aβ fibril formation [41].

On the other hand, the global metabolic analyses identified PGs as the main group whose abundance was decreased, specifically in compounds PGs 44:12, 40:7, and 36:2. However, the role of PGs in AD is still not very clear. A recent publication identified altered metabolites in the cerebral cortices of an Aβ_1-42_-induced rat model of AD, and only two specific PGs were found altered when comparing AD and control models. Specifically, PG 18:1/18:2 and PG 42:6 were downregulated and upregulated, respectively, in group AD compared with group sham (control) [42]. In another study, potential lipidic biomarkers in blood plasma from patients with dementia diseases, including AD, were studied, confirming that only one PG was significantly decreased in AD [43]. Nevertheless, PG is a precursor of cardiolipin (which content and FA composition have been confirmed to be altered in AD [44]), so it is understandable that these changes could be caused by alterations in the PG profile. A recent study has also shown that some PG species (18:2/16:0, 18:2/16:1, 18:2/18:1, 18:2/18:2, 18:2/20:3, and 18:2/20:4) with FAs mainly found in cardiolipin are increased in a cellular AD model in comparison to a control cell [11]. Based on those studies, our results suggest that the decrease of some PGs together with the increase of PUFAs could be related with the neuroprotective potential of the DS extract against the different AD toxic agents.

In summary, and considering all the metabolomics results, we could confirm that the pre-treatment with DS extract is able to alter the lipid metabolism of SH-SY5Y cells, and we hypothesize that the increase of PC content in these cells caused by the extract could have a partial neuroprotective effect against the neurotoxic agents tested, although further in vivo studies should be carried out to confirm this hypothesis.

Finally, to expand the knowledge on the mechanism of action of DS extract in SH-SY5Y, the extracellular medium was also analyzed in terms of carotenoid composition. The data obtained from the extracellular medium composition suggested that the main identified carotenoids are not differentially removed from the cell culture medium by the SH-SY5Y cells, since no significant differences were observed in the ratios 24 h/0 h between plates with and without cells. Additionally, results indicated that carotenoid levels were altered in time, not caused by cell metabolism but by cell culture conditions (24 h, 37 °C, 5% CO_2_). It has been demonstrated that carotenoids are susceptible to spontaneous chemical oxidation and degradation during incubation in cell culture environment, leading to cleavage products such as β-apocarotenoids, which also have potent biological activity [45]. On the other hand, it is well known that the bioavailability of pure carotenoids is limited due to their non-polar nature and, as humans are not able to synthesize them, essential carotenoids such as β-carotene, α-carotene, and β-cryptoxanthin (precursors of vitamin A) must be incorporated through digestion processes [46]. These complex processes may transform them into more bioavailable compounds that could reach the brain after crossing the blood–brain barrier [47].

Furthermore, as other studies have demonstrated, it is possible that some DS extract’s carotenoids present in the extracellular medium (such as cryptoxanthin, zeaxanthin, or lutein) could bind Aβ peptides, preventing the Aβ assembly and, thus, protecting neurons from neurotoxicity [48]. Therefore, it is suggested that DS extract can exert intracellular changes in lipidomic composition of SH-SY5Y, possibly through extracellular interactions between DS extract compounds and cell membrane, and/or by incorporating other minor compounds not detected through HPLC–DAD-MS/MS analysis.

## Conclusions

In the present work, a comprehensive in vitro multi-target study of the neuroprotective potential of *Dunaliella salina* extract, enriched in carotenoids, has been accomplished. Biological activities such as antioxidant, anti-inflammatory, and anti-cholinergic activities of the extract were confirmed, as well as its neuroprotective effect against the neurotoxic agents Aβ_1-42_ and l-glutamate in a neuron-like cell model. Furthermore, advanced bioinformatics and statistical tools allowed the identification of more than 314 metabolites in SH-SY5Y cells, of which 50 lipids were found significantly altered by the DS extract. More specifically, a great number of phosphatidylcholines, triacylglycerols, and fatty acids were significantly increased, while several phosphatidylglycerols were decreased. This fact, along with the possible role exerted by carotenoids and other minor compounds on the cell membrane and on the chemical structure of the neurotoxic agents, might explain the observed neuroprotective effect of the DS extract. However, future experiments using in vivo models to corroborate this hypothesis must be carried out.

## Supplementary Information

Below is the link to the electronic supplementary material.

Supplementary file1 (DOCX 627 KB)

Supplementary file2 (XLSX 10 KB)

Supplementary file3 (XLSX 19 KB)

Supplementary file4 (XLSX 95 KB)

Supplementary file5 (XLSX 35 KB)

Supplementary file6 (XLSX 10 KB)
