# Supplementary material for: Study of the potential neuroprotective effect of Dunaliella salina extract in SH-SY5Y cell model
Source: Anal Bioanal Chem. 2021 Dec 18;414(18):5357–71. doi: 10.1007/s00216-021-03819-1 (PMC9242911; doi:10.1007/s00216-021-03819-1)
Supplement: Supplementary file 1 — Supplementary file1 (DOCX 627 KB) [file 216_2021_3819_MOESM1_ESM.docx]

**Supplementary Information to:**

**Article title:** Study of the potential neuroprotective effect of *Dunaliella* *salina* extract in SH-SY5Y cell model.

**Jounal name**: Analytical and Bioanalytical Chemistry

**Authors name**: Rocío Gallego ^1&^, Alberto Valdés ^1&^*, José D. Sánchez-Martínez ^1^, Zully J. Suárez-Montenegro ^1^, Elena Ibáñez ^1^, Alejandro Cifuentes ^1^ and Miguel Herrero ^1^

^1^ Laboratory of Foodomics, Institute of Food Science Research (CIAL, CSIC-UAM), Calle Nicolás Cabrera 9, 28049 – Madrid, Spain.

* Corresponding author: a.valdes@csic.es; Tel.: +34-910017821

^&^ These two authors contribute equally to this paper

**FIGURE CAPTIONS**

**Fig. S1** Effect of different concentrations of *D. salina* (DS) extract on cell viability in HK-2 cells (2.5 to 40 µg/mL) and differentiated SH-SY5Y cells (10 to 20 µg/mL). Results (mean ± standard deviation) are shown as percentage of living cells compared to control (C; only ethanol-treated). *Denotes statistical differences between the control (C) and a specific DS extract concentration (*: p < 0.05)

**Fig. S2** Correlation plots of retention time (RT) and fold change (FC) values of commonly annotated lipids by CSH-Q-TOF MS/MS (+) and CSH-Q-TOF MS/MS (-); and annotated polar metabolites by HILIC-Q-TOF MS/MS (+) and HILIC-Q-TOF MS/MS (-). The correlations are presented by Pearson’s correlation coefficients (r)

**Fig. S3** PCA and PLS-DA score plots of data obtained by CSH-Q-TOF MS/MS in both ionization modes, after incubation of SH-SY5Y cells with DS extract at 20 μg/mL compared to control conditions for 24 h. (A) PCA ESI (+); (B) PCA ESI (-); (C) PLS-DA ESI (+); (D) PLS-DA ESI (-)

**Fig. S4** PCA and PLS-DA score plots of data obtained by HILIC-Q-TOF MS/MS in both ionization modes, after incubation of SH-SY5Y cells with DS extract at 20 μg/mL compared to control conditions for 24 h. (A) PCA ESI (+); (B) PCA ESI (-); (C) PLS-DA ESI (+); (D) PLS-DA ESI (-)


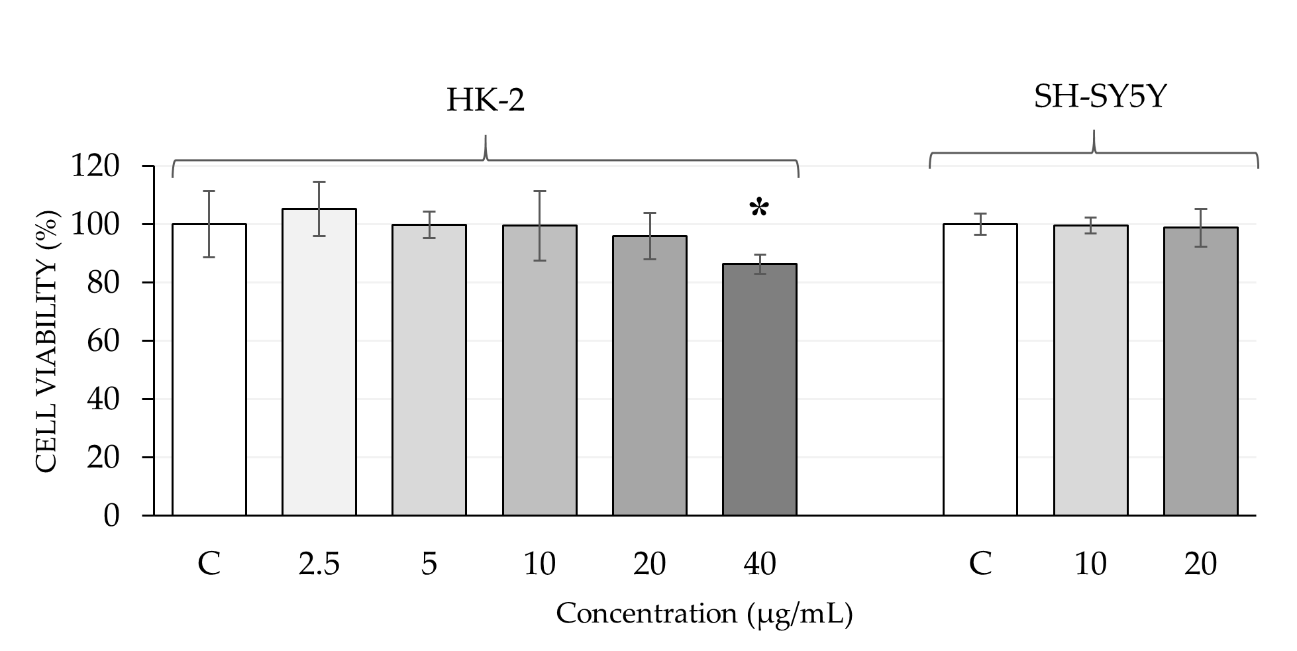


**Fig. S1**


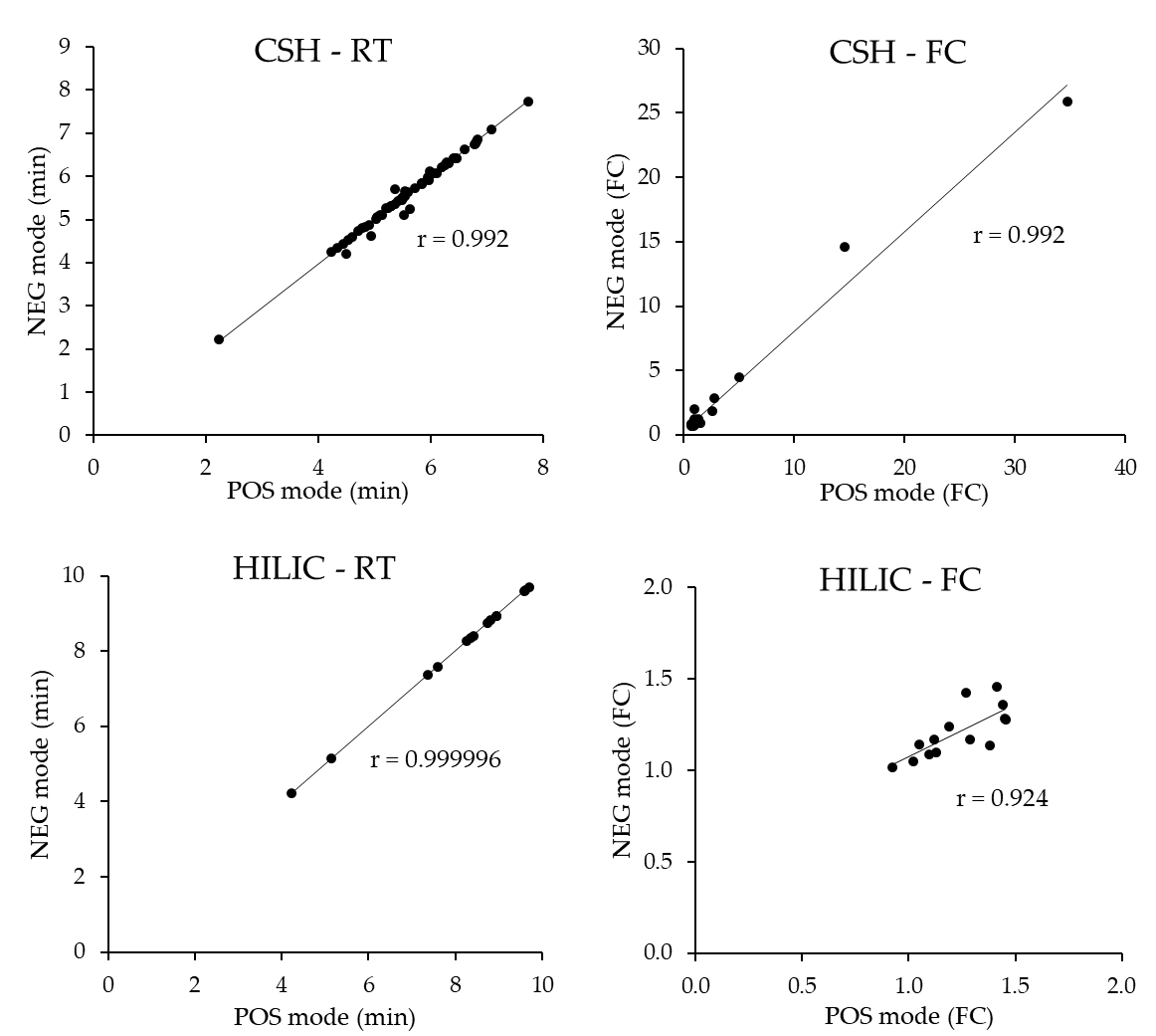


**Fig. S2**

**
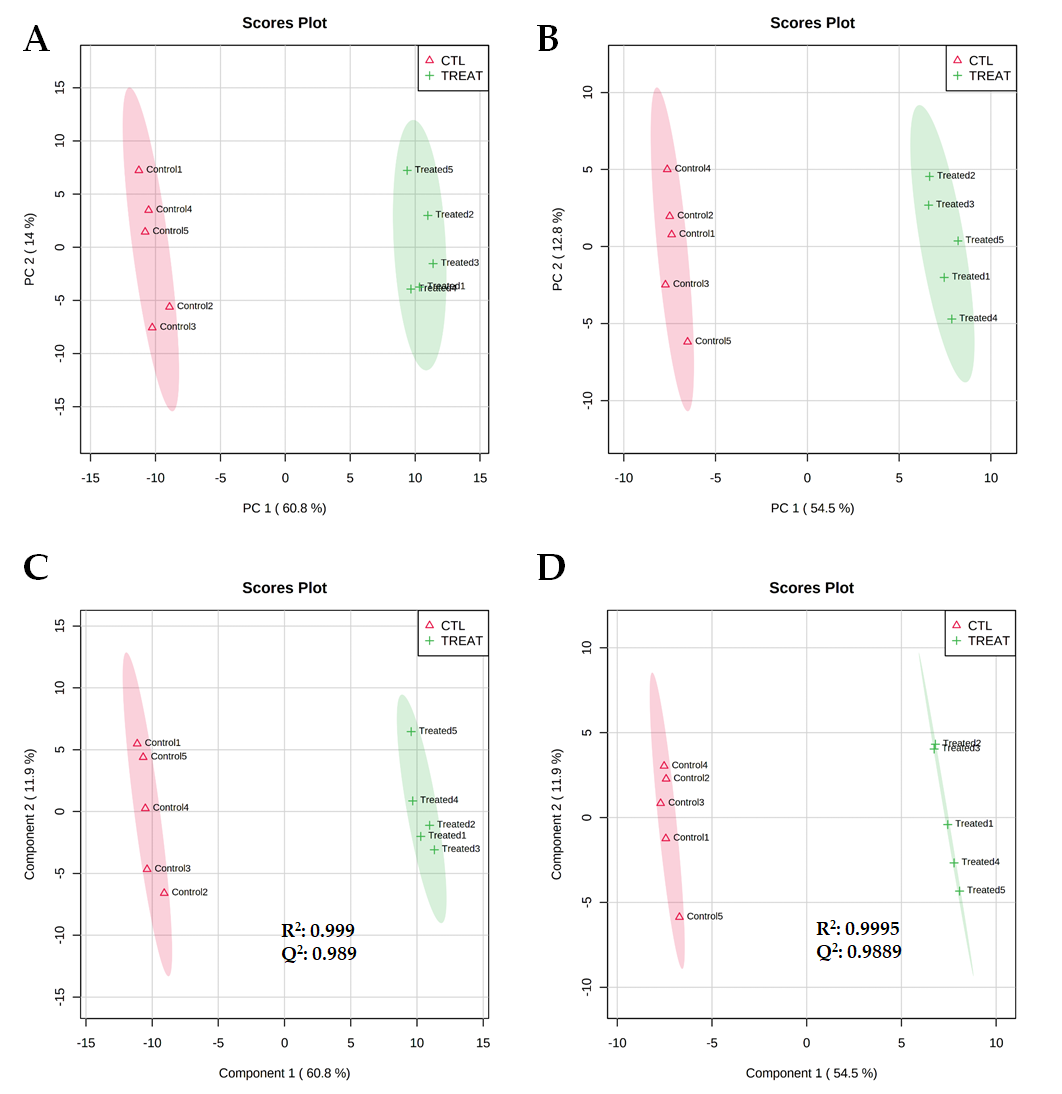
**

**Fig. S3**


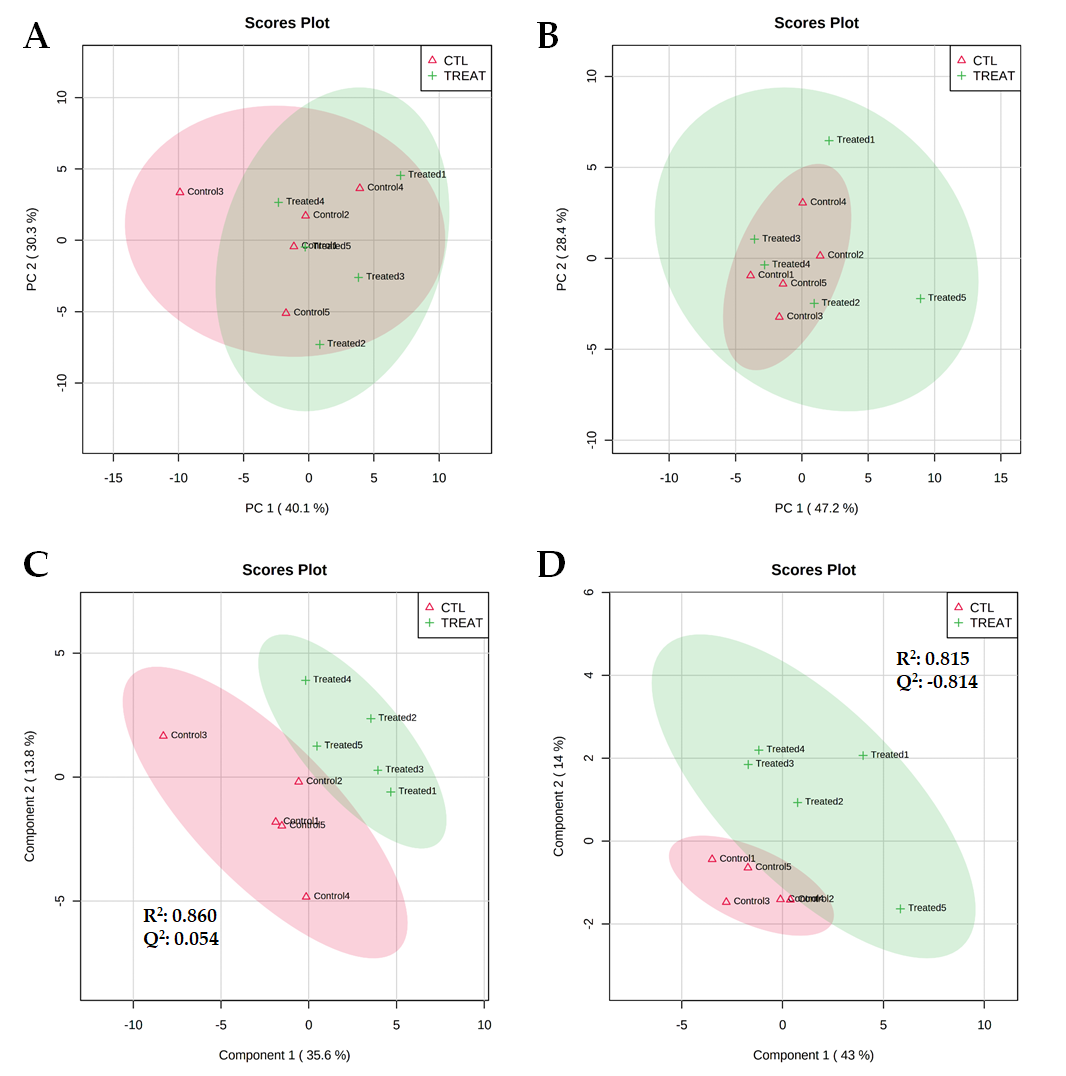


**Fig. S4**

**TABLE LEGENDS**

*Tables are uploaded independently as ESM_TableSX.*

**Table S1.** Concentration of internal standards used during sample preparation.

**Table S2.** Relative standard deviation of the internal standards included during sample preparation and obtained from CSH-Q-TOF MS/MS (ESI (+) and ESI (-)) and, HILIC-Q-TOF MS/MS (ESI (+) and ESI (-)).

**Table S3.** Lists of annotated lipids in CSH-Q-TOF MS/MS (+) and (-) data from SH-SY5Y cells after incubation with DS extract at 20 μg/mL compared to control conditions for 24 h, and including the statistical values for U test and PLS-DA. Cells in green show FC < 0.666, in red FC > 1.5. Values of FDR < 0.05 are shown in blue.

**Table S4.** Lists of annotated metabolites in HILICQ-TOF MS/MS (+) and (-) data from SH-SY5Y cells after incubation with DS extract at 20 μg/mL compared to control conditions for 24 h, and including the statistical values for U test and PLS-DA. Cells in green show FC < 0.666, in red FC > 1.5.

**Table S5.** ChemRICH results obtained after comparing SH-SY5Y cells incubated with DS extract at 20 μg/mL to control conditions for 24 h.
